# A PSO-Based Hybrid Metaheuristic for Permutation Flowshop Scheduling Problems

**DOI:** 10.1155/2014/902950

**Published:** 2014-01-29

**Authors:** Le Zhang, Jinnan Wu

**Affiliations:** ^1^School of Information Engineering, Shenyang University, Shenyang 110044, China; ^2^School of Information Science and Technology, Tsinghua University, Beijing 100084, China

## Abstract

This paper investigates the permutation flowshop scheduling problem (PFSP) with the objectives of minimizing the makespan and the total flowtime and proposes a hybrid metaheuristic based on the particle swarm optimization (PSO). To enhance the exploration ability of the hybrid metaheuristic, a simulated annealing hybrid with a stochastic variable neighborhood search is incorporated. To improve the search diversification of the hybrid metaheuristic, a solution replacement strategy based on the pathrelinking is presented to replace the particles that have been trapped in local optimum. Computational results on benchmark instances show that the proposed PSO-based hybrid metaheuristic is competitive with other powerful metaheuristics in the literature.

## 1. Introduction

Due to the strong industrial background, the permutation flowshop scheduling problem (PFSP) has attracted considerable attention from researchers all over the world. In this problem, a set of jobs *J* = {1,2,…, *n*} needs to be processed through a set of machines *M* = {1,2,…, *m*}. Each job *i* ∈ *J* should be processed through these *m* machines with the same machine order, that is, starting from machine 1 and finishing on the last machine *m*. The processing time of each job *i* ∈ *J* on machine *j* ∈ *M*(*p*_*ij*_) is nonnegative and known before scheduling. It is assumed that all the jobs are available before processing and once started the processing cannot be interrupted. It is required that each job can only be processed by only one machine at any time, and at the same time each machine cannot process more than one job. The processing of a job cannot start on the next machine *j* + 1 until this job has been completed on the current machine *j* and machine *j* + 1 is idle. The objective is to determine the sequence of these *n* jobs so that a certain performance measure can be optimized. The most commonly studied performance measures are the minimization of makespan (*C*_max⁡_) and the minimization of total flowtime (TFT). Let *π* = (*π*(1),…, *π*(*n*)) denote a permutation of the *n* jobs, in which *π*(*k*) represents the job arranged at the *k*th position, and the completion time of each job *π*(*k*) on each machine *j* can be calculated as follows:

(1) Cπ(1),1=pπ(1),1,Cπ(1),j=Cπ(1),j−1+pπ(1),j, j=2,3,…,m,Cπ(k),1=Cπ(k−1),1+pπ(k),1, k=2,3,…,n,Cπ(k),j=max⁡⁡{Cπ(k),j−1,Cπ(k−1),j}+pπ(k),j,k=2,3,…,n; j=2,3,…,m.

Then the makespan can be defined as *C*_max⁡_(*π*) = *C*_*π*(*n*),*m*_, and the total flowtime can be defined as the sum of completion times of all jobs TFT(*π*) = ∑_*k*=1_^*n*^ *C*_*π*(*k*),*m*_.

Since the first introduction of the PFSP [1], considerable attention of researchers has been paid to this problem and many kinds of algorithms have been proposed in the literature. According to the comprehensive review of Ruiz and Maroto [2] and Framinan et al. [3] for PFSP, these solution methods can be classified into three categories: exact methods, heuristics methods, and metaheuristic methods.

Since it has been proven that the PFSP with makespan minimization is NP-complete in the strong sense when *m* ≥ 3 and the PFSP with total flowtime minimization is NP-complete in the strong sense when *m* ≥ 2 [4], few exact methods have been proposed for PFSP in the literature due to their unacceptable computation time. These exact methods include the mixed integer linear programming method [5] and the branch and bound algorithms ([6–8] for the makespan minimization and [9–11] for the total flowtime minimization). However, these exact methods are feasible for only small size problems because they cannot solve large size problems in reasonable computation time.

Heuristic methods can be classified into two categories: constructive heuristics and improvement heuristics. Constructive methods start from an empty solution and try to build a feasible solution in a short time. Johnson's algorithm [1] is the earliest known heuristic for PFSP, which can obtain optimal solutions for *m* = 2. Campbell et al. [12] proposed the CDS heuristic, and Koulamas [13] proposed a two-phase heuristic for PFSP, which were both extensions of Johnson's algorithm. Palmer [14] proposed a slope index heuristic for PFSP, and then Gupta [15] and Hundal and Rajgopal [16] extended Palmer's heuristic and proposed two simple heuristics. Nawaz et al. [17] proposed a so-called NEH heuristic based on the idea that jobs with high total processing times on all machines should be scheduled as early as possible, and this NEH heuristic is regarded as the best heuristic for PFSP with makespan minimization (Ruiz and Maroto [2], Taillard [18]). Recently, complex heuristics have been proposed for PFSP, for example, Liu and Reeves [19] and Framinan and Leisten [20]. As far as the solution quality is concerned, the FL heuristic proposed by Framinan and Leisten [20] was the best one among simple heuristics (Framinan et al. [3]). Contrary to constructive heuristics, improvement heuristics start from an existing initial solution and try to improve it by a given procedure, for example, a local search. Fan and Winley [21] proposed a heuristic named the intelligent heuristic search algorithm for the PFSP. Suliman [22] proposed a two-phase improvement heuristic, in which an initial solution is generated by the CDS heuristic in the first phase and then improved by a pair exchange neighborhood search in the second phase. Framinan et al. [23] proposed a new efficient heuristic for the PFSP with no-wait constraint.

Metaheuristics are high-level strategies that combine and guide other heuristics in the hope of obtaining a more efficient or more robust procedure so that better solutions can be found. The main procedure of metaheuristics generally starts from an initial solution or a set of solutions generated by heuristics and iterates to improve the initial solution or solutions until a given stopping criterion is reached. The metaheuristics proposed for PFSP are mainly the genetic algorithm (Chang et al. [24], Ruiz et al. [25]), the simulated annealing (Hooda and Dhingra [26], Nouri et al. [27]), the tabu search (Gao et al. [28]), the ant colony algorithm (Rajendran and Ziegler [29]), the iterated greedy algorithm (Ruiz and Stützle [30]), and the particle swarm optimization (PSO) (Tasgetiren et al. [31], Wang and Tang [32]). These metaheuristics use the benchmark problems proposed by Taillard [33] to evaluate their performance. The ant colony algorithms proposed by Rajendran and Ziegler [29], named M-MMAS and PACO, obtained much better solutions than constructive heuristics of Framinan and Leisten [20]. The iterated greedy algorithm proposed by Ruiz and Stützle [30] improved the best known results for some instances of PFSP with makespan minimization. The particle swarm optimization (PSO) named PSO_VNS_, which incorporates variable neighborhood search (VNS) into PSO, proposed by Tasgetiren et al. [31] improved 57 out of 90 best known solutions reported by Framinan and Leisten [20] and Rajendran and Ziegler [29] for the total flowtime criterion.

In this paper, we propose an improved PSO for the PFSP. To enhance the exploration ability of PSO, the path relinking and the hybrid simulated annealing with stochastic VNS are incorporated. To improve the search diversification of PSO, a population update method is applied to replace the nonpromising particles. The rest of this paper is organized as follows.  Section 2 is devoted to describe the proposed PSO algorithm. The computational results on benchmark problems are presented in Section 3. Finally, Section 4 concludes the paper.

## 2. PSO Algorithm for PFSP

### 2.1. Brief Introduction of PSO

PSO algorithm is a population based metaheuristic method introduced by Kennedy and Eberhart [34, 35] based on the social behavior of bird flocking and fish schooling, as well as the means of information exchange between individuals, to solve optimization problems. In the PSO, a swarm consists of *m* particles and these particles fly around in an *n*-dimensional search space. The solution of a problem is represented by the position of a particle; that is, the *i*th particle at the *t*th generation is denoted as *X*_*i*_^*t*^ = [*x*_*i*1_^*t*^, *x*_*i*2_^*t*^,…, *x*_*in*_^*t*^]. At each generation, the flight of each particle is determined by three factors: the inertia of itself, the best position found by itself (*p*_best_), and the best position found by the whole swarm (*g*_best_). Generally, *p*_best_ and *g*_best_ are represented as *P*_*i*_^*t*^ = [*p*_*i*1_^*t*^, *p*_*i*2_^*t*^,…, *p*_*in*_^*t*^] and *G* ^*t*^ = [*g*_1_^*t*^, *g*_2_^*t*^,…, *g*_*n*_^*t*^], respectively. Then the velocity of the particle *V*_*i*_^*t*^ = [*v*_*i*1_^*t*^, *v*_*i*2_^*t*^,…, *v*_*in*_^*t*^] for the next generation can be obtained from the following equation:

(2) $$\begin{array}{c} v_{ij}^{t+1}=w\cdot v_{ij}^{t}+c_{1}r_{1}\cdot \left(p_{ij}^{t}-x_{ij}^{t}\right)+c_{2}r_{2}\cdot \left(g_{j}^{t}-x_{ij}^{t}\right),\\ x_{ij}^{t+1}=x_{ij}^{t}+v_{ij}^{t+1}, \end{array}$$

where *w* is called the *inertia *parameter, *c*_1_ and *c*_2_ are the *cognitive *and *social* parameters, and *r*_1_, *r*_2_ are random numbers between (0,1). Based on the above equations, the particle can fly through search space toward *p*_best_ and *g*_best_ in a navigated way while still exploring new areas by the stochastic mechanism to escape from local optima.

### 2.2. Solution Representation

Since the PSO operates in the continuous space, a job is represented by a dimension of a particle and then the *n* jobs can be denoted as a particle *X*_*i*_^*t*^ = [*x*_*i*1_^*t*^, *x*_*i*2_^*t*^,…, *x*_*in*_^*t*^] in the continuous space. Due to the continuous characters of the position values of particles in the PSO, the smallest position value (SPV) rule proposed by Tasgetiren et al. [31] is adopted to transform a particle with continuous position values into a job permutation. A simple example is provided in Table 1 to show the mechanism of the SPV rule. In this instance (*n* = 9), the smallest position value is *x*_*i*2_^*t*^ = −1.75, so job 2 is assigned to the first position of the job permutation according to the SPV rule; then job 9 is assigned to the second position of the job permutation because it has the second smallest position value *x*_*i*9_^*t*^ = −1.21. With the same way, other jobs are assigned in their corresponding position of the job permutation according to their position values. Thus, based on the SPV rule, the job permutation is obtained; that is, *π*_*i*_^*t*^ = (2,9, 4,3, 5,1, 8,7, 6).

### 2.3. Population Initialization

The population with *n*_pop_ solutions is initialized with random solutions according to *x*_*ij*_^0^ = *x*_min⁡_ + rand × (*x*_max⁡_ − *x*_min⁡_), where rand is a uniform random number in [0, 1], *x*_min⁡_ = −4.0, and *x*_max⁡_ = 4.0. Also, we generate the corresponding velocity of each particle by a similar way: *v*_*ij*_^0^ = *v*_min⁡_ + rand × (*v*_max⁡_ − *v*_min⁡_), where *v*_min⁡_ = −1.0 and *v*_max⁡_ = 1.0. In addition, another solution generated by the NEH heuristic [18] is added to the initial population and replaces a random selected solution so as to ensure the quality of initial population.

### 2.4. Hybrid Method of Simulated Annealing and Stochastic VNS

In the PSO_VNS_ proposed by Tasgetiren et al. [31], a stochastic VNS, which itself is a variant of VNS (Hansen and Mladenović [36]), is developed as the local search. For a given discrete job permutation *π* ^*t*^, let *w* and *z* denote two different random integer numbers generated in [1, *n*], and then the two stochastic neighborhoods moves used in the stochastic VNS to generate a neighbor solution *π* ^*t*′^ are (1)  *π* ^*t*′^ = insert(*π* ^*t*^, *w*, *z*): remove the job at the *w*th position and insert it in the *z*th position; and (2)  *π* ^*t*′^ = interchange(*π* ^*t*^, *w*, *z*): interchange two jobs arranged at the *w*th position and the *z*th position. After a job permutation is changed according to a local search operator such as insert or interchange, the position value of each dimension is adjusted correspondingly to guarantee that the permutation that resulted by the SPV rule for new position values is the same as the permutation that resulted by the local search operator. For example, Table 2 shows the interchange move applied to two jobs 3 and 7, and the corresponding position value changes. It is clear that the interchange of jobs 3 and 7 is corresponding to the interchange of position values −1.02 and 0.23. The position value adjustment for the insert move is similar.

To further improve the exploration ability of the local search, we incorporate the solution acceptance scheme of simulated annealing into the stochastic VNS and thus obtain a hybrid method of simulated annealing and stochastic VNS (denoted as SA_VNS). To reduce the computation time and make the search process focus on the intensification phase, we use a decreasing acceptance threshold to act as the cooling procedure of simulated annealing. The procedure of the proposed SA_VNS algorithm is illustrated in Algorithm 1.

In the PSO_VNS_ proposed by Tasgetiren et al. [31], the stochastic VNS is applied on the global best particle found at each iteration. However, a drawback of such application is that the starting point of the stochastic VNS may be the same solution if the global best particle cannot be improved for a number of consecutive iterations, and consequently the exploration ability of the PSO may be decreased. Therefore, for a given population at iteration *t*, we propose to use the following strategy.

**Step 1**

Apply the SA_VNS on the promising particles satisfying (*f*(*π* ^*t*^) − *f*(*g*_best_))/*f*(*g*_best_) ≤ 0.02, in which *f*(*π* ^*t*^) is the objective value of particle *π* ^*t*^.

**Step 2**

Update the global best particle. If a new global best particle is found, then further improve it using the SA_VNS.

### 2.5. Population Update Method

It is well known that the advantage of PSO is that it has a high convergence speed. However, this advantage may become the disadvantage for complex scheduling problems because the scheduling problems generally have many local optimal regions in the search space. That is, for the PSO applied to PFSP, some particles may always fly around a local region and thus are trapped in local optimum. Therefore, we propose a solution replacement strategy based on the pathrelinking [37] to remove these solutions with new solutions with good quality.

In our algorithm, a particle is viewed as being trapped in local optimum if its personal best solution *p*_best_ has not been improved for a number of consecutive generations (i.e., 20). For these particles, we give them the last chance to stay in the population by applying the path relinking algorithm on it to check if its personal best *p*_best_ can be improved. If so, this particle can remain in the population; otherwise, we replace this particle with a new random particle.

The path relinking is originally proposed by Glover et al. [37] to generate new solutions by exploring a path that connects an *initial solution* and a *guiding solution*. In this path, moves are selected that introduce attributes contained in the guiding solution. To present the path relinking algorithm, we define the distance between two particles *X*_*i*_ and *X*_*j*_ as *d*(*X*_*i*_, *X*_*j*_) = *d*(*π*_*i*_, *π*_*j*_) = |*h*(*π*_*i*_) − *h*(*π*_*j*_)|, where *π* is the corresponding job permutation (obtained by the SPV rule) of particle *X* and *h*(*π*) is the Hash function value of *π* = (*π*(1),…, *π*(*n*)) that is calculated as *h*(*π*) = ∑_*k*=1_^*n*^ *k* × *π*(*k*) × *π*(*k*). Then the path relinking algorithm can be described as follows.

**Step 1**

Set *π*_*i*_ = (*π*_*i*_(1), *π*_*i*_(2),…, *π*_*i*_(*n*)) as the *initial solution*. Find the particle that has the largest distance to particle *X*_*i*_ in the current population (denoted as *X*_*j*_), and set its corresponding job permutation *π*_*j*_ = (*π*_*j*_(1), *π*_*j*_(2),…, *π*_*j*_(*n*)) as the *guiding solution*. Set *k* = 1 and the local optimum *π*_opt_ = *π*_*i*_.

**Step 2**

If *π*_*i*_(*k*) ≠ *π*_*j*_(*k*), find the job with index *π*_*j*_(*k*) in *π*_*i*_ and swap it with *π*_*i*_(*k*) to generate a new job permutation *π*_*i*_′. If *π*_*i*_′ is better than *π*_opt_, then set *π*_opt_ = *π*_*i*_′.

**Step 3**

Set *k* = *k* + 1. If *k* > *n* − 10, stop; otherwise, go to Step 2.

It should be noted that the above path relinking stops when *k* is larger than *n* − 10, because if particle *X*_*j*_ is better than *X*_*i*_, then *X*_*i*_ will be replaced by *X*_*j*_ if the path relinking stops when *k* = *n*, which may result in duplicated particles and thus decrease the search diversification.

### 2.6. Complete Procedure of the Proposed PSO

**Step 1**

*Initialization*

*Step 1* *.1*. Set initial values for the population size *n*_pop_, the *inertia* weight, and the *cognitive* and *social* parameters. Set *t* = 0 and *NO*_*i*_ = 0  (*i* = 1,2,…, *n*_pop_) for each particle in the population. Create the initial population and the initial velocities for each particle using the method described in Section 2.3.

*Step 1.2*. Generate the job permutation for each particle in the population using the SPV rule, and calculate the objective value of each particle.

*Step 1.3*. Set the personal best of each particle to be the particle itself and the global best to be the best one among the population.

**Step 2**

*Update Particle Positions*

*Step 2.1*. Update iteration counter *t* = *t* + 1.

*Step 2.2*. Update *inertia* weight by *w* = *w* × *β*.

*Step 2.3*. For each particle, update the velocity and position values according to (2).

*Step 2.4*. Generate the job permutation for each particle in the current population using the SPV rule, and calculate the objective value of each particle.

**Step 3**

*Local Search Phase*

*Step 3.1*. Use the SA_VNS algorithm to improve the promising particles in the current population and then the global best particle found so far according to the adoption strategy of SA_VNS described in Section 2.4.

*Step 3.2*. For each particle in the current population, update its personal best *p*_best_. If *p*_best_ of particle *i* is improved, then set *NO*_*i*_ = 0; otherwise set *NO*_*i*_ = *NO*_*i*_ + 1.

**Step 4**

*Population Update*. For each particle in the current population, use the population update method described in Section 2.5 to update the current population.

**Step 5**

*Stopping Criterion*. If *t* > *T*_max⁡_ (the maximum iteration number) or the runtime has reached the limit, then stop; otherwise, go to Step 2.

## 3. Computational Experiments

To test the performance of our PSO algorithm (denoted as PSO*), computational experiments were carried out on the well-known standard benchmark set of Taillard [33] that is composed of 110 instances ranging from 20 jobs and 5 machines to 200 jobs and 20 machines. This benchmark set contains some instances proven to be very difficult to solve. In this benchmark set there are 10 instances for each problem size. Our PSO algorithm was implemented using C++ and tested on a personal PC with Pentium IV 3.0 GHz CPU and 512 MB memory. To make a fair comparison with the PSO_VNS_, we use the same parameter setting proposed by Tasgetiren et al. [31]. That is, the population size is taken as *n*_pop_ = 2*n*; the initial inertia weight is set to *w* = 0.9 and never less than 0.4; the decrement factor *β* for *w* is taken as 0.975; the acceleration coefficients are set to *c*_1_ = *c*_2_ = 2; the maximum iteration number *T*_max⁡_ is taken as 500.

### 3.1. Results for PFSP with Makespan Minimization

For the makespan minimization objective, our PSO algorithm was compared with other powerful methods, for example, the ant colony algorithm named PACO of Rajendran and Ziegler [29], the genetic algorithm named HGA_RMA of Ruiz et al. [25], the iterated greedy algorithm named IG_RS_LS_ of Ruiz and Stützle [30], and the PSO_VNS_ algorithm of Tasgetiren et al. [31]. The solution quality was measured by the average relative percent deviation (denoted as ARPD) over *R* replicated runs for each instance in makespan with respect to the best known upper bounds. More specifically, ARPD is calculated as ARPD = ∑_*i*=1_^*R*^(((*H*_*i*_ − *U*_*i*_) × 100)/*U*_*i*_)/*R*, in which *H*_*i*_ is the makespan obtained by a certain algorithm, whereas *U*_*i*_ is the best known upper bound value for Taillard's instances as of April 2004 for the makespan criterion. As done by many researchers, *R* is set to *R* = 10 in our experiments.

The comparison results for these algorithms are given in Table 3, in which the values are the average performance of the 10 instances for each problem size. As seen in Table 3, our PSO* algorithm achieves the best average performance and it obtains the best results for instances of 20 × 5, 20 × 10, 50 × 5, 50 × 10, 100 × 5, 100 × 20, and 200 × 20. The IG_RS_LS_ method also performs well with the HGA_RMA method being close. More specifically, the PACO method cannot obtain the lowest ARPD for any group of problem size compared to other rival methods. The HGA_RMA method has the lowest ARPD for instances of 20 × 10, 50 × 5, and 100 × 10. The IG_RS_LS_ method demonstrates the best results for instances of 20 × 20, 50 × 5, 50 × 10, 50 × 20, and 200 × 10. For instances of 20 × 10, both the HGA_RMA method and the PSO_VNS_ method give the best performance. For instances of 50 × 5, all the four methods except for PACO can obtain the lowest ARPD. For instances of 100 × 5, only the two PSO algorithms give the best performance. Our PSO* algorithm performs better than its rivals in 100 × 20 and 200 × 20 instances, which have been proven more difficult to solve. Therefore, it can be concluded that our PSO* algorithm is competitive with other powerful methods in the literature.

### 3.2. Results for PFSP with Total Flowtime Minimization

For the total flowtime minimization objective, our PSO algorithm was compared with other powerful methods, for example, the constructive heuristics of Framinan and Leisten [20], the ant colony algorithm of Rajendran and Ziegler [29], and the PSO_VNS_ of Tasgetiren et al. [31], using the benchmark problems of Taillard [33]. The solution quality was measured by the relative percent deviation (denoted as RPD) of the best solution found among *R*  (*R* = 5) replicated runs for each instance in the total flowtime criterion with respect to the best known results. That is, RPD is calculated as RPD = min⁡⁡{((*H*_*i*_ − *U*_*i*_) × 100)/*U*_*i*_,  *i* ∈ *R*}, in which *H*_*i*_ is the total flowtime value obtained by a certain algorithm, whereas *U*_*i*_ is the best result obtained among the algorithms of Framinan and Leisten [20] and Rajendran and Ziegler [29] (this best result is denoted as LR and RZ).

For the minimization of the total flowtime criterion, the PSO_VNS_ algorithm [31] is demonstrated to be a very powerful PSO algorithm because it improved 57 out of 90 best known solutions reported in [20, 29]. The comparison results between our PSO* and the PSO_VNS_ are given in Table 4. From this table, we can see that the PSO_VNS_ algorithm can obtain the best results for instances of 20 × 5, 20 × 10, 50 × 5, 100 × 5, and 100 × 10, while our PSO* algorithm obtains the best results for the other large size instances of 20 × 20, 50 × 10, 50 × 20, and 100 × 20. On average, our PSO* shows a much better performance in the solution quality and robustness than the PSO_VNS_ algorithm.

## 4. Conclusions

This paper presents a PSO-based hybrid metaheuristic for the permutation flowshop problems to minimize the makespan and the total flowtime. In this algorithm, a hybrid method of simulated annealing and stochastic variable neighborhood search is incorporated to improve the exploitation ability, and a solution replacement strategy based on the path relinking method is developed to improve the exploration ability. Computational experiments are carried out to test the performance of the proposed PSO-based hybrid metaheuristic, and the results show that the proposed algorithm is competitive or superior to some other powerful algorithms in the literature for this problem. Future research may lie in the application of this algorithm in practical production scheduling problems.

## Figures and Tables

**Algorithm 1:**
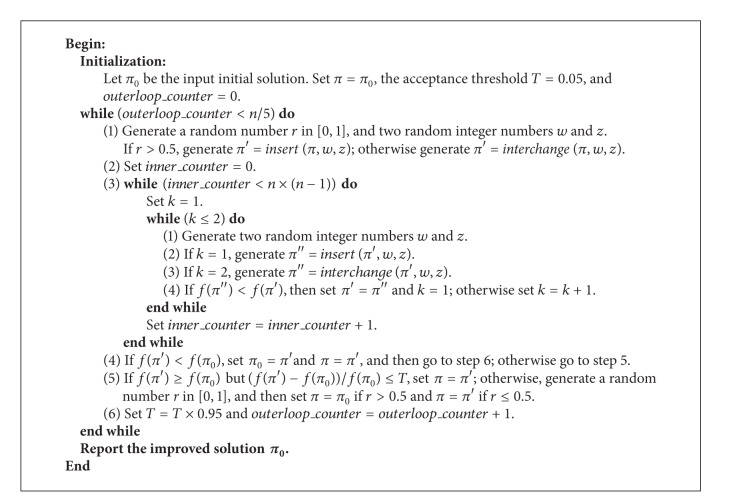
The main procedure of the* SA_VNS*.

**Table 1:** Solution representation and the corresponding job permutation using SPV rule.

Dimension *j*	1	2	3	4	5	6	7	8	9
*x*_*ij*_^*t*^	0.35	−1.75	−0.02	−0.21	0.02	1.20	1.03	0.67	−1.21
Job, π_*ij*_^*t*^	6	1	4	3	5	9	8	7	2

**Table 2:** Interchange move on the job permutation and the corresponding position value adjustment.

Dimension *j*	1	2	3	4	5	6	7	8
Before interchange								
*x*_*ij*_^*t*^	0.54	−0.75	−***1.02***	−0.41	0.92	−1.20	***0.23***	0.12
Job, π_*ij*_^*t*^	6	***3***	2	4	8	***7***	1	5
After interchange								
*x*_*ij*_^*t*^	0.54	−0.75	***0.23***	−0.41	0.92	–1.20	−***1.02***	0.12
Job, π_*ij*_^*t*^	6	***7***	2	4	8	***3***	1	5

The bold and italic values are used to show the interchange move applied to jobs 3 and 7.

**Table 3:** *ARPD* comparison of different methods for makespan criterion.

Problem	PACO	HGA_RMA	IG_RS_LS_	PSO_VNS_	PSO*
20 × 5	0.18	0.04	0.04	0.03	**0.00**
20 × 10	0.24	**0.02**	0.06	**0.02**	**0.02**
20 × 20	0.18	0.05	**0.03**	0.05	0.05
50 × 5	0.05	**0.00**	**0.00**	**0.00**	**0.00**
50 × 10	0.81	0.72	**0.56**	0.57	**0.56**
50 × 20	1.41	0.99	**0.94**	1.36	0.99
100 × 5	0.02	0.01	0.01	**0.00**	**0.00**
100 × 10	0.29	**0.16**	0.20	0.18	0.20
100 × 20	1.93	1.30	1.30	1.45	**1.18**
200 × 10	0.23	0.14	**0.12**	0.18	0.18
200 × 20	1.82	1.26	1.26	1.35	**1.16**
Average	**0.65**	**0.43**	**0.41**	**0.47**	**0.39**

The bold font is used to highlight the better solutions.

*used to denote our algorithm.

**Table 4:** Performance comparison of PSO_VNS_ and PSO* for total flowtime criterion.

Problem	PSO_VNS_		PSO*	
	RPD	CPU (s)	RPD	CPU (s)
20 × 5	**−0.175**	3.18	−0.168	8.11
20 × 10	**−0.037**	7.21	−0.035	4.86
20 × 20	2.758	11.93	**−0.068**	24.97
50 × 5	**−0.603**	41.71	−0.531	40.28
50 × 10	−0.819	74.49	**−0.892**	44.32
50 × 20	0.857	143.32	**−0.543**	50.67
100 × 5	**−0.570**	222.28	−0.546	409.75
100 × 10	**−0.692**	407.88	−0.636	414.14
100 × 20	−0.104	824.41	**−0.801**	442.37
Average	**0.068**	**192.93**	**−0.469**	**159.941**

The bold font is used to highlight the better solutions.

*used to denote our algorithm.
